# The Histone Acetyltransferase *HpGCN5* Involved in the Regulation of Abiotic Stress Responses and Astaxanthin Accumulation in *Haematococcus pluvialis*

**DOI:** 10.3389/fpls.2022.903764

**Published:** 2022-05-20

**Authors:** Danqiong Huang, Wenfu Liu, Qunju Hu, Hui Li, Chaogang Wang

**Affiliations:** ^1^Shenzhen Key Laboratory of Marine Bioresource and Eco-Environmental Science, Shenzhen Engineering Laboratory for Marine Algal Biotechnology, Guangdong Provincial Key Laboratory for Plant Epigenetics, College of Life Sciences and Oceanography, Shenzhen University, Shenzhen, China; ^2^Marine Resources Big Data Center of South China Sea, Southern Marine Science and Engineering Guangdong Laboratory, Zhanjiang, China

**Keywords:** *Haematococcus pluvialis*, *HpGCN5*, abiotic stresses, correlation network, astaxanthin

## Abstract

The histone acetyltransferases (HATs), together with histone deacetylases, regulate the gene transcription related to various biological processes, including stress responses in eukaryotes. This study found a member of HATs (*HpGCN5*) from a transcriptome of the economically important microalgae *Haematococcus pluvialis*. Its expression pattern responding to multiple abiotic stresses and its correlation with transcription factors and genes involved in triacylglycerols and astaxanthin biosynthesis under stress conditions were evaluated, aiming to discover its potential biological function. The isolated *HpGCN5* was 1,712 bp in length encoding 415 amino acids. The signature domains of Acetyltransf_1 and BROMO were presented, as the GCN5 gene from *Arabidopsis* and *Saccharomyces cerevisiae*, confirming that *HpGCN5* belongs to the GCN5 subfamily of the GNAT superfamily. The phylogenetic analysis revealed that *HpGCN5* is grouped with GNAT genes from algae and is closer to that from higher plants, compared with yeast, animal, fungus, and bacteria. It was predicted that *HpGCN5* is composed of 10 exons and contains multiple stress-related *cis*-elements in the promoter region, revealing its potential role in stress regulation. Real-time quantitative PCR revealed that *HpGCN5* responds to high light and high salt stresses in similar behavior, evidenced by their down-regulation exposing to stresses. Differently, *HpGCN5* expression was significantly induced by SA and Nitrogen-depletion stresses at the early stage but was dropped back after then. The correlation network analysis suggested that *HpGCN5* has a strong correlation with major genes and a transcription factor involved in astaxanthin biosynthesis. Besides, the correlation was only found between *HpGCN5* and a few genes involved in triacylglycerols biosynthesis. Therefore, this study proposed that *HpGCN5* might play a role in the regulation of astaxanthin biosynthesis. This study firstly examined the role of HATs in stress regulation and results will enrich our understanding of the role of HATs in microalgae.

## Introduction

Eukaryotes have a complex gene expression regulation mechanism to adapt to developmental and environmental changes, by activation or repression of target genes through chromatin remodeling which is generally regulated by histone modification, DNA methylation, and nucleosome remodeling (Kadonaga, 1998; Loidl, 2004; Pfluger and Wagner, 2007). Acetylation is considered as the most important type of histone modification, which is accomplished by histone acetyltransferases (HATs) and histone deacetylases (HDACs), compared with methylation, phosphorylation, etc. (Kuo and Allis, 1998). It has been recorded that HATs can neutralize the positive charges on lysine residues through acetylation to loosen the chromatin, which facilitates the binding of transcriptional regulatory proteins to DNA and thereby promotes gene transcription (Roth et al., 2001). Besides, HATs also can acetylate other non-histone components, such as transcription factors, transcription co-regulators, DNA-binding proteins, and non-nuclear proteins, to regulate the gene expression (Panagopoulos et al., 2001).

According to the amino acid sequence characterization, HATs can be classified into four groups, namely, the CBP family (p300/CREB-binding protein), GNAT (GCN5-related N-terminal acetyltransferases)/MYST superfamily, TAFII250 family, and the mammals-unique HAT family (Pandey et al., 2002). Compared with other three families, the GNAT/MYST superfamily has been widely studied. It has been reported that the difference between GNAT and MYST is that there are four motifs in the HAT domain in GNAT members, while only one in MYST members (Sterner and Berger, 2000). Previous studies divided GNAT members into four subfamilies, namely, GCN5, ELP3, HAT1, and HAT2 (Roth et al., 2001). In *Arabidopsis*, the homolog of each subfamily was found in the genome, except for the HAT2 subfamily (Pandey et al., 2002). Later, experiments confirmed that the members of GNAT play an important role in plant growth, development, and stress responses (Stockinger et al., 2001; Vlachonasios et al., 2003; Chinnusamy and Zhu, 2009; Fang et al., 2014; Hu, 2015; Ueda and Seki, 2020).

In the microalgae, histone modification has been reported in *Ostreococcus tauri, Ostreococcus lucimarinus, Bathycoccus prasinos*, and *Micromonas sp*., in the manner of methylation (Kim et al., 2015). However, HATs have been rarely reported in microalgae. To date, only a gene *CrGNAT* encoding the acetyltransferase in *Chlamydomonas reinhardtii* has been reported to regulate the responses to heavy metals (Wang et al., 2015). As an economically important microalga, *Haematococcus pluvialis* is popular for its superior biosynthesis of astaxanthin, which is a super antioxidant (Shah et al., 2016). In general, the production of astaxanthin is commonly induced by stress conditions in *H. pluvialis* (Shah et al., 2016). Even though the fact of histone modification playing an important role in the stress response has been widely demonstrated in plants (Ueda and Seki, 2020), the fundamental research on histone modification in stress responses in *H. pluvialis* is still not carried out. In this study, the candidates of histone acetyltransferases were isolated and characterized in *H. pluvialis*, aiming to have a first view of the role of HATs in abiotic stress responses and its potential role in astaxanthin and triacylglycerols biosynthesis. The results will potentially extend the understanding of the mechanism of stress-induced astaxanthin biosynthesis.

## Materials and Methods

### Algae Culture and Growth Conditions

*Haematococcus pluvialis* strain 192.80 used in this study was from Experimental Phycology and Culture Collection of Algae (EPSAG), Goettingen University (Goettingen, Germany). Algal cells were cultured in a 250 ml flask with 100 ml ESP basal medium with peptone as recommended by EPSAG (https://www.uni-goettingen.de/en/186449.html), in a growth chamber under continuous illumination (25 μmol photon m^−2^s^−1^) at 22°C, with gently shaking by hand daily. At the middle logarithmic growth stage (about 1.5 × 10^5^ cell/ml), algal cells were subjected to four independent abiotic stresses, including, high salt (HS), salicylic acid (SA), high light (HL), and Nitrogen-depletion (N-).

For the HS treatment and SA treatment, filter-sterilized sodium acetate or salicylic acid solution was added into the cultures at the final concentration of 45 mM and 0.18 M, respectively. For the HL treatment, algal cells were exposed to continuous irradiance at 500 μmol photon m^−2^s^−1^. For the N- treatment, algal cells were harvested by mild centrifugation at 1,000 rpm for 10 min and resuspended in the BG11 medium (Allen, 1968) without nitrate components. For the HL and HS stresses, algal cells were treated for 24 h and samples were collected at 0, 1.5, 3, 6, 12, and 24 h. For the SA and N- stresses, algal cells were treated for 5 days and samples were collected daily. For each treatment, a total of 18 cultures with 100 ml in 250 ml flasks were prepared and three cultures were randomly harvested at each collection time as three biological replicates.

### Transcriptomic Identification and Experimental Cloning of *HpGCN5*

An Iso-seq transcriptome database was previously obtained using RNA extracted from *H. pluvialis* grown under the high light stress condition. To identify the potential histone acetyltransferase, a query database was constructed by using histone acetyltransferases recorded in NCBI. TBLASTN program was then performed locally using the query database against the Iso-seq database. The candidates of histone acetyltransferases were screened out using a threshold e-value of 1e-5. Aiming to dissociate the GNAT members from potential histone acetyltransferase, the maximum likelihood phylogenetic tree was conducted by MEGA7 software using their putative proteins and GNAT genes from *Arabidopsis* (GenBank accession: AAK31318.1), *Saccharomyces cerevisiae* (Genbank accession: NP_011768.1), and *Chlamydomonas reinhardtii* (Genbank accession: XP_001693868.1). Finally, the candidates of GNAT in *H. pluvialis* were manually checked by SMART and Pfam analyses to avoid errors normally generated by the large-scale bioinformatic analysis. Hence, the candidate of *GCN5* belonging to the GNAT family would be identified based on the domain analysis.

Thereby, the candidate of the GCN5 gene (HpGCN5) was experimentally isolated from *H. pluvialis* by RT-PCR and T-A cloning strategy. For the RT-PCR, the cDNA template was obtained from the RNA used for the Iso-seq database construction, with the help of Perfect Real-Time PrimeScript™ RT Reagent Kit with gDNA Eraser (TaKaRa, China). Gene-specific primers used to isolate the target gene were designed based on its nucleotides from the Iso-seq database (GF1: 5′-GACACTAGGAGGACATCAGGACAAAT-3′; GR1: 5′-GTGTACCAACAAGCGACTGCGACT-3′). The RT-PCR reaction consisted of 1 μl cDNA, 1.0 μl each forward and reverse primer (10 μM), 10 μl 2 × Platinum™ SuperFi II Green PCR Master Mix (Invitrogen Life technologies, United States), and 7.2 μl ddH_2_O. The amplification was performed on the Bio-Rad T100 thermal cycler (Bio-Rad, United States) and the condition was as follows: initial denaturation at 98°C for 2 min; 35 cycles of 98°C for 10 s, 60°C for 10 s, 72°C for 1.5 min; and a final extension at 72°C for 5 min. Agarose gel electrophoresis, PCR products purification, T-A cloning including vector construction, *E. coli* transformation, colony PCR identification, and sequencing were performed as previously reported (Huang et al., 2019).

### Bioinformatics Analysis

The ORF and deduced amino acids of isolated *HpGCN5* were carried out by EditSeq (DNASTAR software, Lasergene, United States). To determine the sequence identity and divergence, multiple sequence alignment was performed by MegAlign (DNASTAR software, Lasergene, United States). The domain of deduced amino acid was predicted by SMART online at http://smart.embl-heidelberg.de/. To carry out the potential gene structure of *HpGCN5*, the cDNA sequence was aligned with the genome sequence of *H. pluvialis* (BIG Data Center GSA Database accession no. PRJCA000614) (Luo et al., 2019). The schematic diagram of gene structure was displayed by Gene Structure Display Server 2.0 (http://gsds.cbi.pku.edu.cn/). Based on the genome sequence, the *cis*-elements within 2,000 bp upstream of the transcription start site of *HpGCN5* were searched in the PlantCARE database (http://bioinformatics.psb.ugent.be/webtools/plantcare/html/) (Lescot et al., 2002). The phylogenetic relationship was analyzed by MEGA7 software with the Minimum Evolution method (Kumar et al., 2016).

To evaluate if *HpGCN5* is involved in the stress response, triacylglycerols biosynthesis, and astaxanthin biosynthesis, the correlations between *HpGCN5* and associated genes were analyzed using transcriptome data driven from *H. pluvialis* treated with SAHL (salicylic acid combined with high light) and SAHS (salicylic acid combined with high salt) that were previously reported by our research group (Hu et al., 2021). First, a gene cluster was constructed, including *HpGCN5* and 310 differentially expressed transcripts (49 were annotated as transcription factors, 169 were associated with carotenoids biosynthesis, and 92 were associated with triacylglycerols biosynthesis). The expression data of the gene cluster were retrieved from the SAHL and SAHS transcriptome data (NCBI SRA database accession no. PRJNA675306), respectively. Finally, the Pearson statistical analysis was performed at a significance of 0.05, and the correlation network was constructed using genes with a correlation >0.8.

### Expression Pattern Analyses Responding to Various Abiotic Stresses

The quantitative real-time PCR (qRT-PCR) was performed to evaluate the expression pattern of *HpGCN5* responding to various abiotic stresses in this study. For each sample collected from each time point of each stress treatment, total RNA was exacted by RNA fast 200 Kit (Fastagen, China) and the first strand of cDNA was synthesized by PrimeScript™ RT reagent Kit with gDNA Eraser (Perfect Real-Time) (TaKaRa, China), according to the corresponding user instruction. The qRT-PCR reaction consisted of 6 μl TB Green™ Premix Ex Taq ™II (Tli RNaseH Plus) (TaKaRa, China), 1 μl cDNA, 0.5 μl each forward and reverse primer (10 μM), and 4 μl ddH_2_O. The amplification was as follows: initial denaturation at 95°C for 30 s, 40 cycles of 95°C for 5 s, and 60°C for 30 s. The amplification was monitored on an ABI QuantStudio™ 6 Flex System (Applied Biosystems, United States). To quantify the expression level of *HpGCN5* in each sample, *HpActin* was employed as an internal reference (Wen et al., 2015). Primers targeting *HpGCN5* were qGF (5′-ATCATGCACGGCCTCTCCCACAGC-3′) and qGR (5′-GACTTCAGCCGACCCCTCCTTCAG-3′), and targeting *HpActin* were qAF (5′-AGCGGGAGATAGTGCGGGACA-3′) and qAR (5′-ATGCCCACCGCCTCCATGC-3′), respectively. The expression level of *HpGCN5* in each sample was calculated by the 2^−Δ*ΔCt*^ method (Mackay et al., 2002). All reactions were run in triplicate.

### Statistical Analysis

Expression levels of *HpGCN5* between each sample collected from the same abiotic stress were compared by Pearson's *t*-test. The significance was determined at the level of 0.05, 0.01, and 0.001.

## Results

### Transcriptome-Wide Identification of GCN5 Candidates

Based on the local TBLASTN using NCBI-recorded histone acetyltransferase from various species, 52 transcripts were hit. Since some transcripts shared the same amino acids, their putative amino acids were used for further analysis, instead of nucleotides. It was found that 52 transcripts were associated with 26 genes, which were subjected to the phylogenetic analysis. Results suggested that two genes, namely, Gene005060 and Gene010338, were clustered with *AtGCN5* and *ScGCN5* from *Arabidopsis* and *S. cerevisiae*, respectively, while Gene002197 was clustered with CrGNAT from *C. reinhardtii* (Supplementary Figure S1). Subsequently, these three candidates were subjected to further SMART analysis to figure out the possible protein domains. Results suggested that all three candidates contained the signature Acetyltransf_1 domain (Pfam accession: PF.00583), the same as AtGCN5, ScGCN5, and CrGNAT did, indicating that these three candidates should be the members of the GNAT family (Supplementary Figure S2). Furthermore, two of them (Gene005060 and Gene010338) contained the additional BROMO domain (Pfam accession: PF00439), as *AtGCN5* and *ScGCN5* did. Hence, Gene005060 and Gene010338 were selected as the candidates for *HpGCN5* genes.

### Experimental Cloning and Sequence Characterization of *HpGCN5*

**Figure 1:**
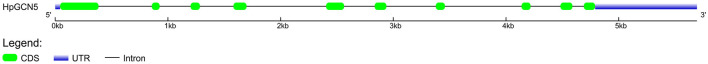
The schematic diagram of *HpGCN5* gene structure, which was predicted by aligning with the corresponding genome sequence.

**Figure 2:**
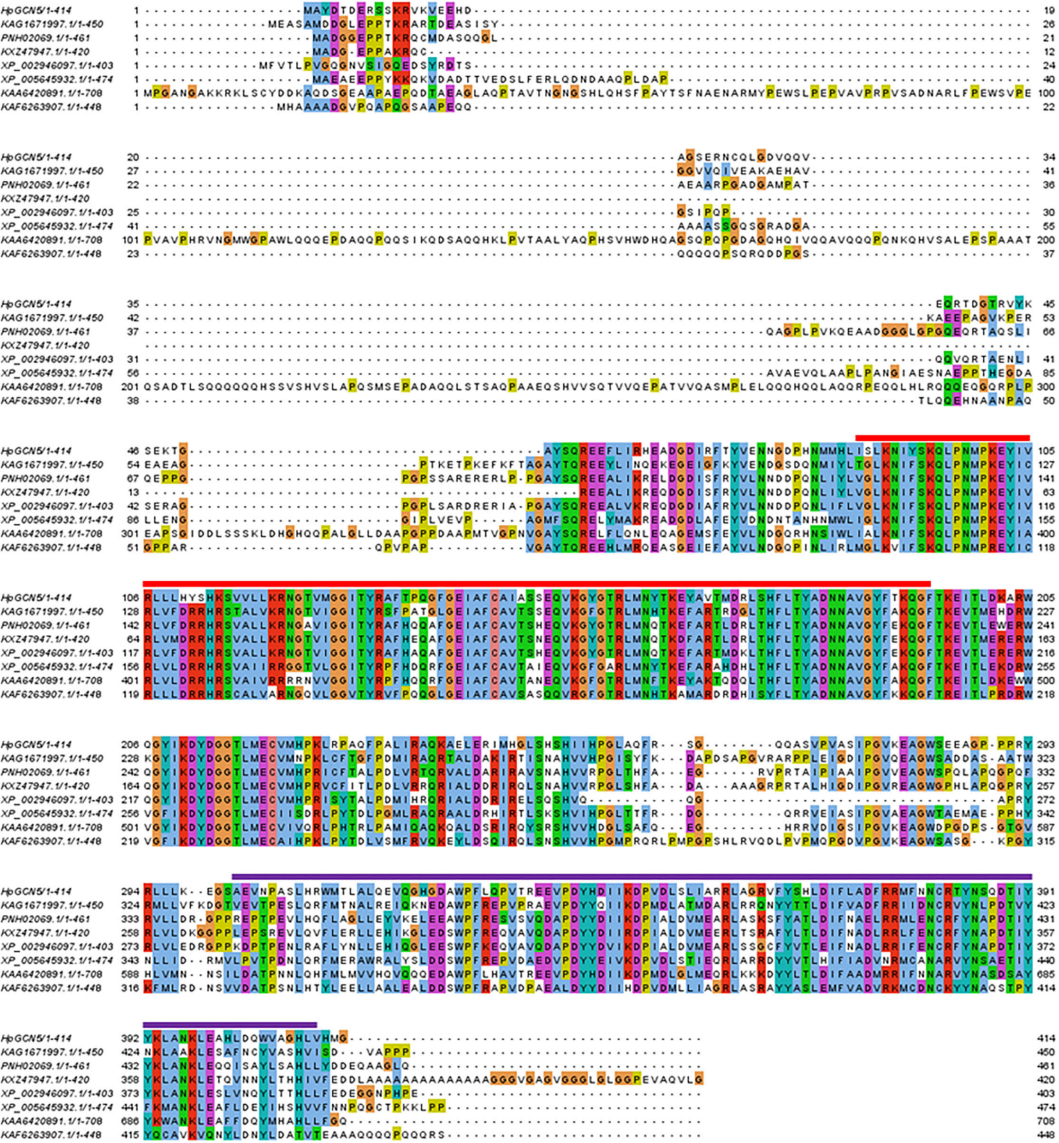
The alignment of amino acids of *GCN5* gene from *Haematococcus pluvialis* and its homologous from other species including KAG1671997.1 from *Chlamydomonas sp*., PNH02069.1 from *Tetrabaena socialis*, KXZ47947.1 from *Gonium pectoral*, XP002946097.1 from *Volvox carteri*, XP 005645932.1 from *Coccomyxa subellipsoidea*, KAA6420891.1 from *Trebouxia sp*., and KAF6263907.1 from *Scenedesmus sp*. The Acetyltransf_1 domain (Pfam accession: PF00583) was shown by the red line and the BROMO domain (Pfam accession: PF00439) was shown by the purple line.

Based on previous bioinformatics analysis, Gene005060 and Gene010338 were considered the HpGCN5 candidate genes. However, only Gene010338 was successfully experimental cloned, which was renamed *HpGCN5*. The isolated cDNA sequence of *HpGCN5* was 1,712 bp in length and the open reading frame (ORF) was predicted to be 1,245 bp in length encoding 415 amino acids. By BLASTN against the genome sequence of *Haematococcus pluvialis* (BIG Data Center GSA Database accession no. PRJCA000614) using the *HpGCN5* cDNA sequence, a scaffold was carried out. Based on the alignment, the *HpGCN5* cDNA sequence covered 5,214 bp in the scaffold and was divided into 10 exons (Figure 1, Supplementary Data 1). The putative amino acid was analyzed by SMART and the results turned out two specific domains, Acetyltransf_1 (PF00583) and BROMO (PF00439) were presented indicating that *HpGCN5* should be a member of proteins encoding acetyltransferase. The alignment using putative amino acids suggested high conservation in Acetyltransf_1 and BROMO domain between *HpGCN5* and *GNAT* genes from other algae species, including *Chlamydomonas sp., Tetrabaena socialis, Gonium pectoral, Scenedesmus sp., Coccomyxa subellipsoidea, Trebouxia sp*., and *Scenedesmus sp*. (Figure 2). Meanwhile, *cis*-acting regulatory elements, were predicted in the 2,000 bp upstream of the coding region of *HpGCN5*. Twelve *cis*-elements were carried out, including five involved in light responsiveness (ACE, G-Box, GTGGC-motif, LAMP-element, and Sp1), five involved in small molecular hormone responsiveness (ABRE, CGTCA-motif, TGACG-motif, P-box, and TCA-element), one involved in anaerobic induction (ARE), and one involved in drought inducibility (MBS) (Table 1).

**Table 1:** Stress-related *cis*-elements in the promoter region of *HpGCN5* identified from the PlantCARE database.

***cis*-elements**	**Number of sites**	**Sequence**	**Function**
ABRE	3	ACGTG	Abscisic acid responsiveness
ACE	1	GACACGTATG	Light responsiveness
ARE	1	AAACCA	Anaerobic induction
CGTCA-motif	8	CGTCA	MeJA-responsiveness
TGACG-motif	8	TGACG	MeJA-responsiveness
G-Box	3	CACGTB	Light responsiveness
GTGGC-motif	1	CAGCGTGTGGC	Light responsive element
LAMP-element	1	CCTTATCCA	Light responsive element
MBS	2	CAACTG	Drought-inducibility
P-box	1	CCTTTTG	Gibberellin-responsive element
Sp1	2	GGGCGG	Light responsive element
TCA-element	2	CCATCTTTTT	Salicylic acid responsiveness

### Molecular Evolution of *HpGCN5*

**Figure 3:**
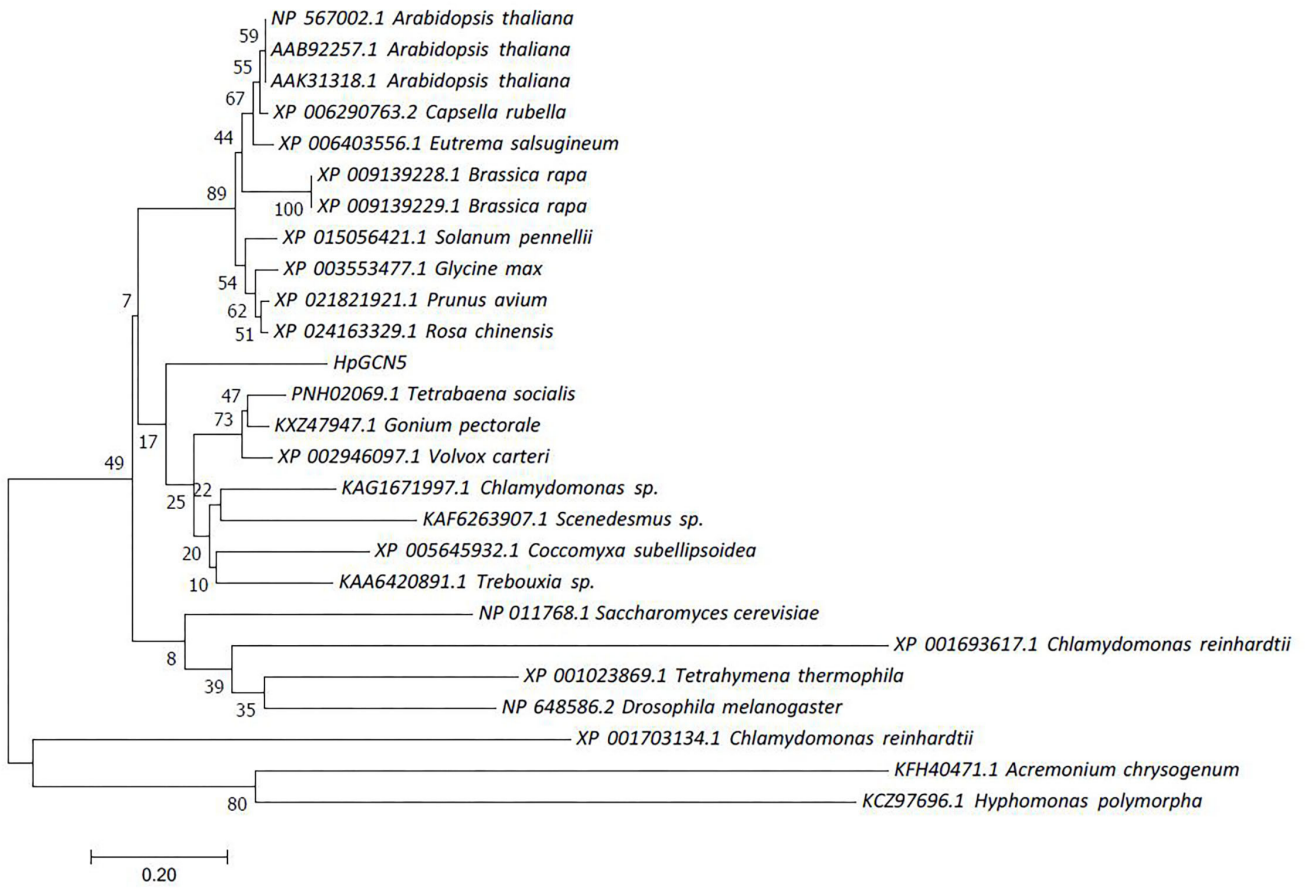
Phylogenetic relationships of the deduced amino acids of *HpGCN5* with its homologous from other species.

To determine the evolutionary pattern of histone acetyltransferase genes, an evolutionary relationship tree was constructed using amino acids obtained in this study and retrieved from GenBank by the Minimum Evolution method. The amino acids used in the tree were from multiple organisms, including algae, higher plants, yeast, animal, fungus, and bacteria (Figure 3). Results found that *HpGCN5* was grouped with most histone acetyltransferase genes from algae, which was distinguished from other species, including higher plants, yeast, animal, fungus, and bacteria. Compared with yeast, animal, fungus, and bacteria, GNAT from algae was closer to higher plants, except XP_001693617.1 and XP_001706134.1 from algae *Chlamydomonas reinhardtii*. Within the algae group, *HpGCN5* found in *H. pluvialis* was dissociated from the other seven algae species.

### Time-Course Expression Profile of *HpGCN5* Under Multiple Abiotic Stresses

**Figure 4:**
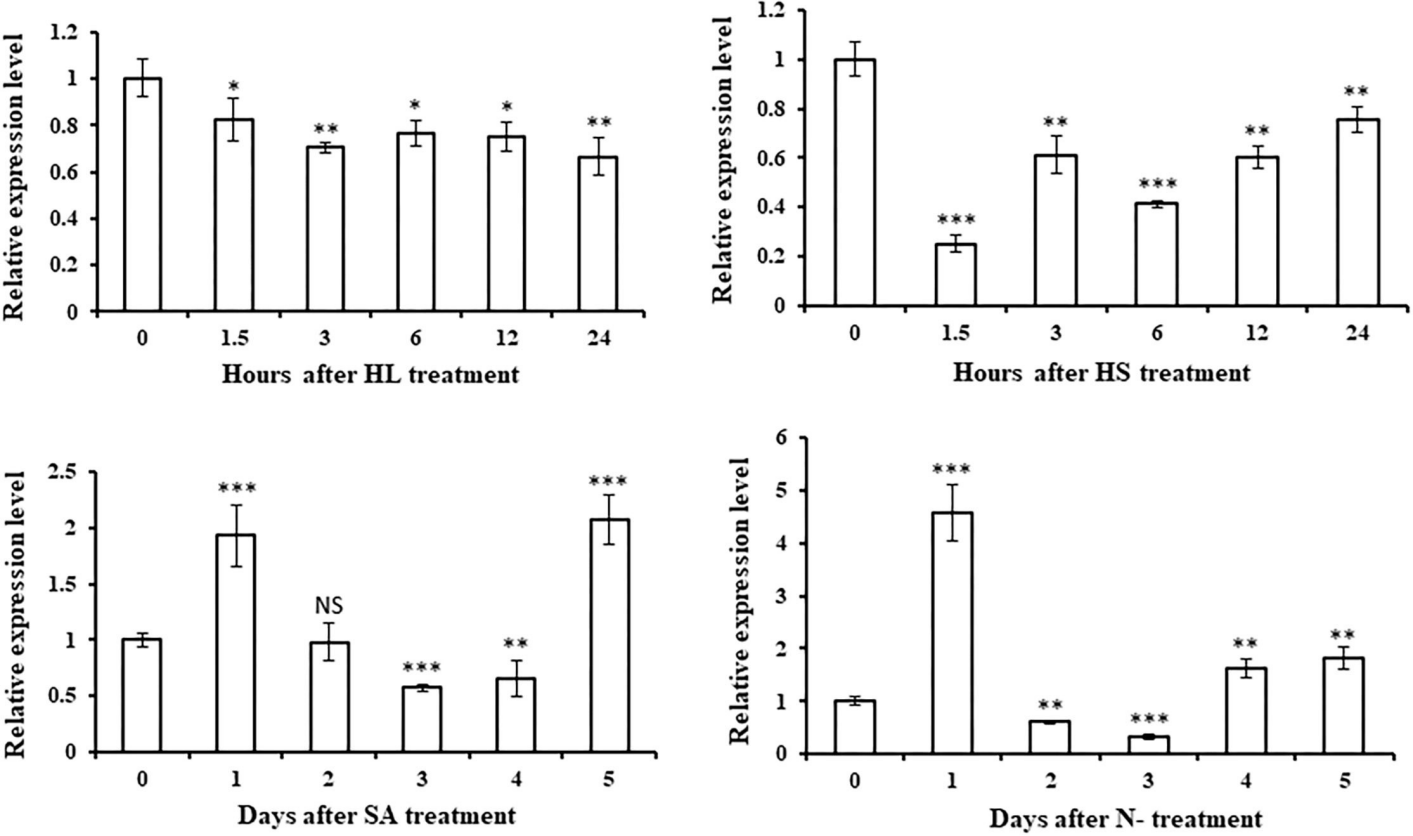
The relative expression levels of *HpGCN5* under multiple abiotic stresses quantified by qRT-PCR assay. HL indicates the high light stress, HS indicates the high salt stress, SA indicates the salicylic acid stress, and N- indicates the Nitrogen-depletion stress. *, **, *** indicates the statistical significance between the treatment and control at the level of 0.05, 0.01, and 0.001, respectively. NS indicates no statistical significance.

To further characterize the biological function of *HpGCN5*, qRT-PCR was carried out to evaluate its expression pattern under multiple stresses in a time-course manner, since stress-related *cis*-elements were present in the promoter region. Expression level changes were observed suggesting that *HpGCN5* should be involved in the stress responses (Figure 4). It was shown that the responses of *HpGCN5* to high light (HL) and high salt (HS) stresses were similar, which was different from that of salicylic acids (SA) and Nitrogen-depletion (N-) stresses. A significant decrease in the transcriptional level of *HpGCN5* was found at the initial stage of HL and HS stresses and the down-regulation was continued in all examined samples. Conversely, a significant increase in the transcriptional level of *HpGCN5* was displayed on Day 1 under SA and N- stresses, respectively. However, the transcriptional level was dramatically dropped back on Day 2 and continued to decrease until Day 3, then started to increase on Day 4 till Day 5. The highest transcriptional level was found on Day 1 under N- stress, which is about 4.5-fold as the control (Day 0).

### Correlation Network of *HpGCN5*

**Figure 5:**
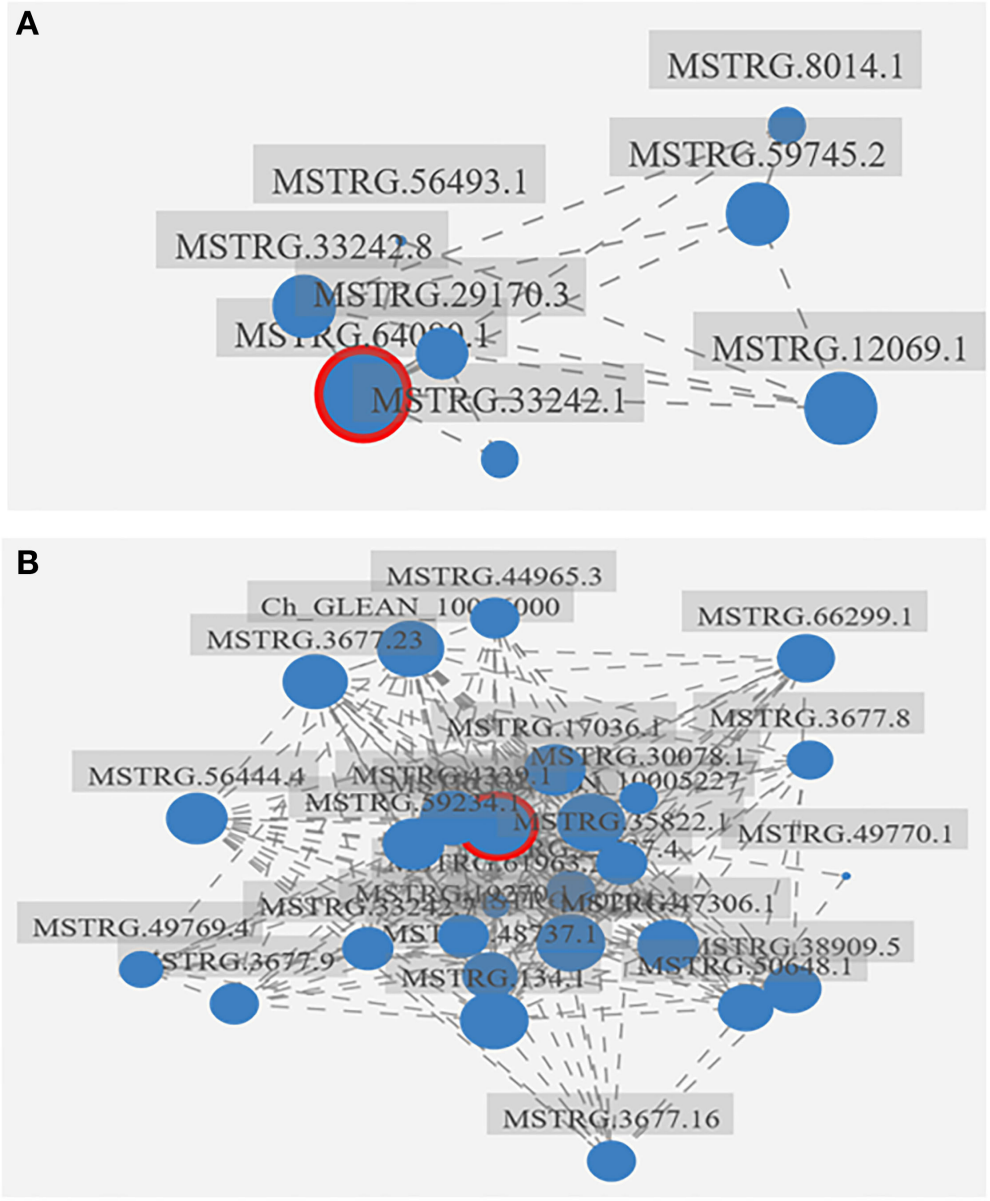
The correlation network of *HpGCN5* and genes involved in carotenoids biosynthesis, triacylglycerols biosynthesis, and transcriptional regulation. **(A)** The correlation network constructed by using SAHL transcriptome data; **(B)** the correlation network constructed by using SAHS transcriptome data. The HpGCN5 was red cycled. The big blue dots indicate the nod in the network, and the bigger the nod, the more correlated transcripts.

To estimate if *HpGCN5* is associated with other genes under stress conditions, the correlation network of *HpGCN5* was constructed. The analyzed targets included 310 differentially expressed transcripts associated with transcription factors, carotenoids, and triacylglycerols biosynthesis identified in *H. pluvialis* grown under SAHL and SAHS conditions. It was found that seven transcripts correlated with *HpGCN5* according to their expression pattern under the SAHL stress condition, whereas 24 transcripts had a correlation with *HpGCN5* under the SAHS stress condition, implying that *HpGCN5* might be more sensitive to SAHS stress than to SAHL stress (Figure 5, Table 2). In detailed, the seven transcripts in SAHL transcriptome included four transcripts associated with carotenoids biosynthesis, two transcripts associated with triacylglycerols biosynthesis, and one transcript involved in transcriptional regulation. The highest correlation factor was −0.957231, which was between *HpGCN5* and MSTRG.12069.1 (a transcript involved in triacylglycerols biosynthesis). The 24 transcripts in SAHS transcriptome included 15, 4, and 5 associated with carotenoids, triacylglycerols, and transcriptional regulation, respectively. The highest factor was −0.994516, which was between *HpGCN5* and Ch_GLEAN_10006000 (a transcript involved in carotenoids biosynthesis). It is interesting that transcripts involved in triacylglycerols biosynthesis were negatively correlated with *HpGCN5* under SAHL stress, but positively correlated with *HpGCN5* under SAHS stress. It indicated that either *HpGCN5* or triacylglycerols biosynthesis genes respond to SAHL and SAHS stress in a different way.

**Table 2:** The annotation of transcripts presented in the correlation network constructed using transcriptome data of SAHL and SAHS, respectively.

**Database**	**Transcript ID**	**Correlation factor**	**Gene annotation**	**Involved in**
SAHL	MSTRG.33242.1	0.816066	Phytoene synthase; ubiquitin carboxyl-terminal	Carotenoids biosynthesis
	MSTRG.56493.1	0.839965	RWP-RK domain-containing transcription factor	Transcriptional regulation
	MSTRG.29170.3	0.905598	Cytochrome P450, carotenoid hydroxylase	Carotenoids biosynthesis
	MSTRG.33242.8	0.934788	Phytoene synthase; ubiquitin carboxyl-terminal	Carotenoids biosynthesis
	MSTRG.8014.1	−0.916011	Phytoene desaturase	Carotenoids biosynthesis
	MSTRG.59745.2	−0.952188	Diacylglycerol acyltransferase	Triacylglycerols biosynthesis
	MSTRG.12069.1	−0.957231	Glycerol-3-phosphate dehydrogenase	Triacylglycerols biosynthesis
SAHS	MSTRG.19270.1	0.820992	Diacylglycerol acyl transferase	Triacylglycerols biosynthesis
	MSTRG.48737.1	0.824332	Squalene/ phytoene synthase	Carotenoids biosynthesis
	MSTRG.30078.1	0.828012	Transcription factor VIP1	Transcriptional regulation
	MSTRG.61963.2	0.828265	Transcription factor MYBC1	Transcriptional regulation
	MSTRG.35822.1	0.858899	Glycerol-3-phosphate acyltransferase	Triacylglycerols biosynthesis
	MSTRG.4339.1	0.891892	Glycerol-3-phosphate dehydrogenase	Triacylglycerols biosynthesis
	MSTRG.47306.1	0.910431	Glycerol-3-phosphate dehydrogenase	Triacylglycerols biosynthesis
	MSTRG.17036.1	0.912888	Squalene/phytoene synthase	Carotenoids biosynthesis
	MSTRG.59234.1	0.913444	Protein phosphatase inhibitor	Transcriptional regulation
	MSTRG.60204.4	0.964641	Phytoene desaturase	Carotenoids biosynthesis
	Ch_GLEAN_10005227	0.964641	Transcription factor GATA	Transcriptional regulation
	MSTRG.134.1	0.964641	Squalene/ phytoene synthase	Carotenoids biosynthesis
	MSTRG.49770.1	−0.811928	Beta-carotene ketolase	Carotenoids biosynthesis
	MSTRG.44965.3	−0.817036	Phytoene desaturase	Carotenoids biosynthesis
	MSTRG.3677.16	−0.829524	Lycopene beta cyclase	Carotenoids biosynthesis
	MSTRG.3677.8	−0.839354	Lycopene beta cyclase	Carotenoids biosynthesis
	MSTRG.66299.1	−0.868443	Beta-carotene hydroxylase	Carotenoids biosynthesis
	MSTRG.38909.5	−0.871466	Beta-carotene hydroxylase	Carotenoids biosynthesis
	MSTRG.3677.9	−0.871688	Lycopene beta cyclase	Carotenoids biosynthesis
	MSTRG.50648.1	−0.877279	Beta-carotene ketolase	Carotenoids biosynthesis
	MSTRG.3677.23	−0.899852	Lycopene beta cyclase	Carotenoids biosynthesis
	MSTRG.56444.4	−0.912418	Transcription factor MYBC1	Transcriptional regulation
	MSTRG.49769.4	−0.91799	Beta-carotene ketolase	Carotenoids biosynthesis
	Ch_GLEAN_10006000	−0.994516	Beta-carotene hydroxylase	Carotenoids biosynthesis

## Discussion

*Haematococcus pluvialis* is a green microalga that naturally accumulates astaxanthin, which is a superior antioxidant with excellent commercial value (Shah et al., 2016). It has been documented that the biosynthesis and accumulation of astaxanthin is an internal stress response to remove free radicals produced when grown under unfavorable conditions (Shah et al., 2016). However, the molecular mechanism of stress responses in *H. pluvialis* is unclear. In higher plants, it has been reported that the histone modification, including histone acetylation, plays an important role in the stress response (Sterner and Berger, 2000; Stockinger et al., 2001; Pfluger and Wagner, 2007; Chinnusamy and Zhu, 2009; Fang et al., 2014; Hu, 2015; Ueda and Seki, 2020). Unfortunately, the discovery and functional analysis of histone modification in microalgae are rare.

In this study, a gene encoding histone acetyltransferase was identified from the Iso-seq transcriptome database. Its responses to multiple abiotic stresses were evaluated and its correlation with transcription factors and genes involved in triacylglycerols and astaxanthin biosynthesis was predicted, aiming to discover its potential role in stress regulation. Based on the bioinformatics analysis, three candidates were found to have the signature Acetyltransf_1 domain in GNAT genes while two of them contained the BROMO domain in GCN5 genes replying there might have two candidates of *HpGCN5*. A previous study found that various organisms, including *Arabidopsis, S. cerevisiae, S. pombe, D. melanogaster, C. elegans*, and even human beings, only have a single representative of GCN5 (Pandey et al., 2002). The experimental isolation was only success in Gene010338 (*HpGCN5*) but failed in Gene005060. A BLASTN has been performed using Gene005060 against the genome sequence of *H. pluvialis*. Results suggested the existence of an intron with 3,287 bp (data not shown), which might challenge the reality of Gene005060. It is well known that the technique of Iso-seq using single-module long reads reduced the accuracy of nucleotides for long transcripts (Gonzalez-Garay, 2016), leading to the possibility that Gene005060 might be a false prediction during transcriptome data processing when using the genome of *H. pluvialis* as the reference to correct the nucleotides. Therefore, we might conclude that *H. pluvialis* also only have a single representative of GCN5, as other species do.

According to the sequence analysis, *HpGCN5* contained 10 exons encoding 415 amino acids (Figure 1). The domain analysis revealed that *HpGCN5* contained a single Acetyltransf_1 and a single BROMO domain (Figure 2) that further assigned *HpGCN5* into the subgroup of GCN5 belonging to the GNAT/MYST superfamily of HATs. In *Arabidopsis*, a single BROMO domain was found in BRD, CHR, HAF, GTE, and HAG1 proteins, leading to the prediction that plants do not have multi-bromodomain proteins like fungi and animals (Pandey et al., 2002). Similarly, a single BROMO domain was also found in *HpGCN5*. Therefore, *HpGCN5* might evolutionarily be close to higher plants than fungi and animals and this was further confirmed by the phylogenetic relationship analysis (Figure 3). Within the microalgae clade in the phylogenetic tree, it showed that *HpGCN5* was dissociated from the other seven algae species. Even though both Acetyltransf_1 and BROMO domain were presented in the other seven algae species and shared high amino acids similarity among species, large variation was found in the non-domain region (Figure 2), leading to the prediction that *H. pluvialis* might have a different ancestor of *HpGCN5* from other algae species.

To explore the biological function of *HpGCN5*, the *cis*-element in the promoter and expression patterns under stress conditions were investigated. It was found that there were 12 *cis*-elements and 10 of them are associated with stress responsiveness, including light and hormones (Table 1). Therefore, the transcriptional expression levels of *HpGCN5* under high light and salicylic acid stress were evaluated. Results turned out high light inhibited while salicylic acid-induced the expression of *HpGCN5* (Figure 4). In *H. pluvialis*, researchers indicated that except high light and hormones, other stress conditions, such as salinity, nitrogen starvation, high temperature, and additional iron supplement, could also accelerate the accumulation of astaxanthin (Raman and Ravi, 2011; Gao et al., 2012a,b, 2015; Hong et al., 2015; He et al., 2018; Zhao et al., 2020; Hu et al., 2021). Therefore, the responses of *HpGCN5* to high salt and Nitrogen-depletion were also evaluated. It is interesting that the high salt stress also inhibited the expression of *HpGCN5* as high light did, whereas, the Nitrogen-depletion treatment generated similar expression changes of *HpGCN5* as salicylic acid did (Figure 4). It is possible that the histone acetylation process controlled by *HpGCN5* should be similar when induced by high light and high salt and it is different from that by hormones and Nitrogen-depletion. It is further revealed that the mechanism of high light and high salt response should be different from hormones and Nitrogen-depletion at the histone modification level.

With the advantage of previously constructed transcriptome data using *H. pluvialis* grown under SAHL and SAHS stress conditions for different periods, this study evaluated the possibility of *HpGCN5* involved in the astaxanthin and triacylglycerol formation, as well as in the transcriptional regulation responding to stress conditions. The formation of astaxanthin and triacylglycerol is a common phenotypical response when grown under stress conditions in *H. pluvialis* (Shah et al., 2016). Meanwhile, the transcription factor has commonly played an important role in gene expression regulation (Hobert, 2008). Hence, the genes involved in carotenoids and triacylglycerols formation and transcriptional regulation were evaluated for their correlation with *HpGCN5*. It turns out major genes in the astaxanthin biosynthesis pathway, including phytoene desaturase (PDS), lycopene beta cyclase (LCY), beta-carotene hydroxylase (CrtR), and beta-carotene ketolase (BKT), were negatively correlated with *HpGCN5* with factors over 0.8 (Figure 5, Table 2). It is surprising that only a few genes involved in triacylglycerols biosynthesis showed a correlation with *HpGCN5* (Figure 5, Table 2), even though the triacylglycerols biosynthesis is also a responsive phenotype for *H. pluvialis* grown under stress conditions (Shah et al., 2016). Furthermore, MYB transcription factors in *H. pluvialis* should play an important role in astaxanthin accumulation by regulating astaxanthin synthesis-related genes (Gao et al., 2015; Wang et al., 2021). This study observed a strong correlation between *HpGCN5* and *MYBC1* (Table 2). Therefore, it might predict that *HpGCN5* indirectly regulated the synthesis of astaxanthin by directly regulating MYB transcription factors. More research can be conducted to further clarify the mechanism of astaxanthin accumulation regulated by *HpGCN5*.

## Data Availability Statement

The datasets presented in this study can be found in online repositories. The names of the repository/repositories and accession number(s) can be found in the article/Supplementary Material.

## Author Contributions

DH, HL, and CW contributed to the conception, design of the study, and edited the manuscript. DH and WL performed experiments. DH and QH analyzed the data. DH wrote the draft of the manuscript. All authors contributed to the article and approved the submitted version.

## Funding

This work was funded by the Natural Science Foundation of Guangdong Province (2019A1515011701) and the Shenzhen Scientific Project (JCYJ20210324093604011).

## Conflict of Interest

The authors declare that the research was conducted in the absence of any commercial or financial relationships that could be construed as a potential conflict of interest.

## Publisher's Note

All claims expressed in this article are solely those of the authors and do not necessarily represent those of their affiliated organizations, or those of the publisher, the editors and the reviewers. Any product that may be evaluated in this article, or claim that may be made by its manufacturer, is not guaranteed or endorsed by the publisher.

## Supplementary Material

Supplementary Figure S1 Maximum Likelihood analysis of *GCN5* candidates from *Haematococcus pluvialis* and reported genes from *Arabidopsis* (AAK31318.1), *Saccharomyces cerevisiae* (NP 011768.1), and *Chlamydomonas reinhardtii* (XP_001693868.1).

Supplementary Figure S2 The domain of *GCN5* candidates from *Haematococcus pluvialis* and reported genes from *Arabidopsis* (AAK31318.1), *Saccharomyces cerevisiae* (NP 011768.1), and *Chlamydomonas reinhardtii* (XP_001693868.1) according to SMART analysis.

Supplementary Data 1 The corresponding genome sequence of *HpGCN5* downloaded from the BIG Data Center GSA Database accession no. PRJCA000614.
