# Supplementary figures and images for: The Histone Acetyltransferase HpGCN5 Involved in the Regulation of Abiotic Stress Responses and Astaxanthin Accumulation in Haematococcus pluvialis
Source: Front Plant Sci. 2022 May 20;13:903764. doi: 10.3389/fpls.2022.903764 (PMC9163953; doi:10.3389/fpls.2022.903764)

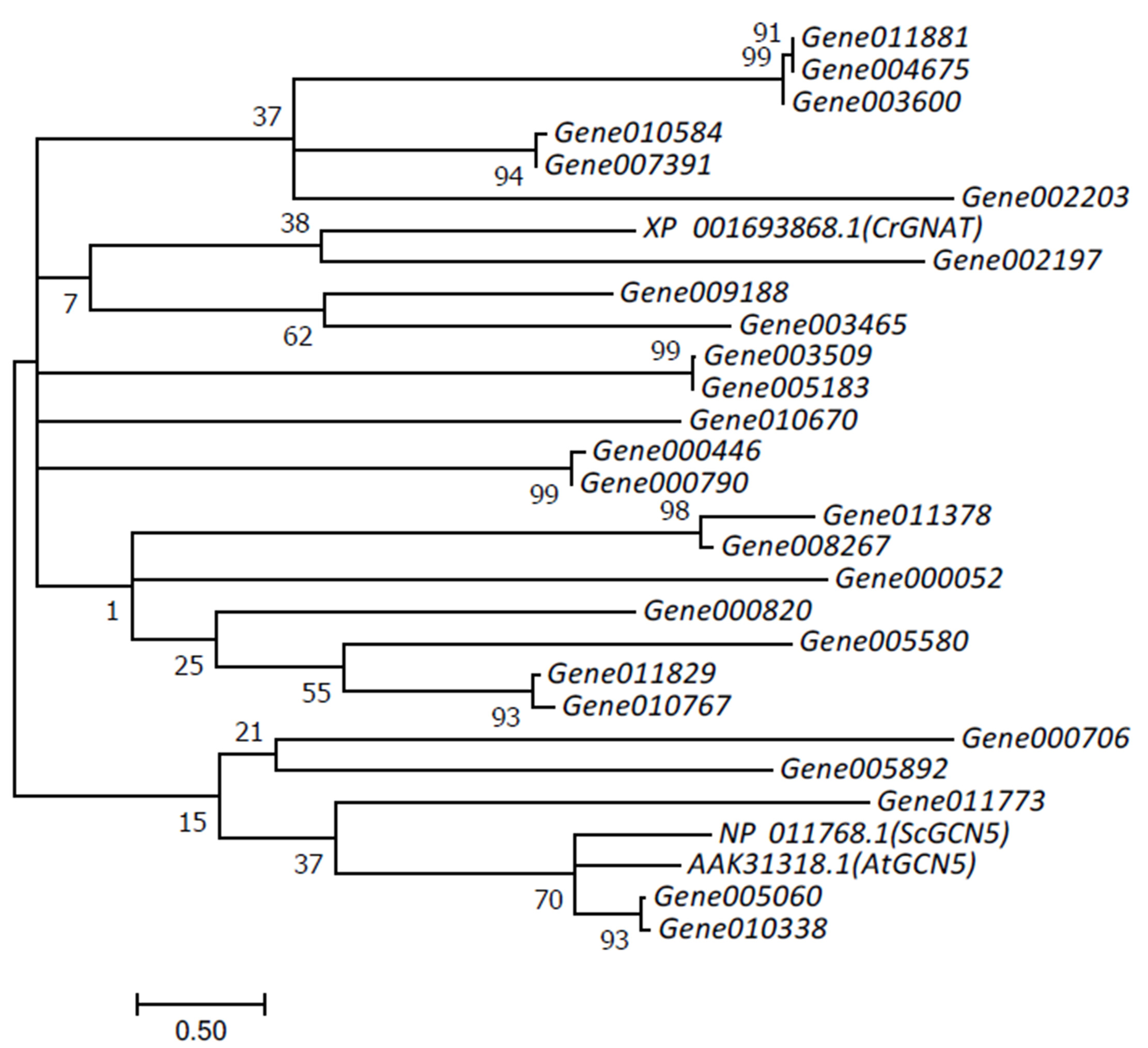

Supplement: Supplementary Figure S1 — Maximum Likelihood analysis of GCN5 candidates from Haematococcus pluvialis and reported genes from Arabidopsis (AAK31318.1), Saccharomyces cerevisiae (NP 011768.1), and Chlamydomonas reinhardtii (XP_001693868.1). [file Image_1.JPEG]

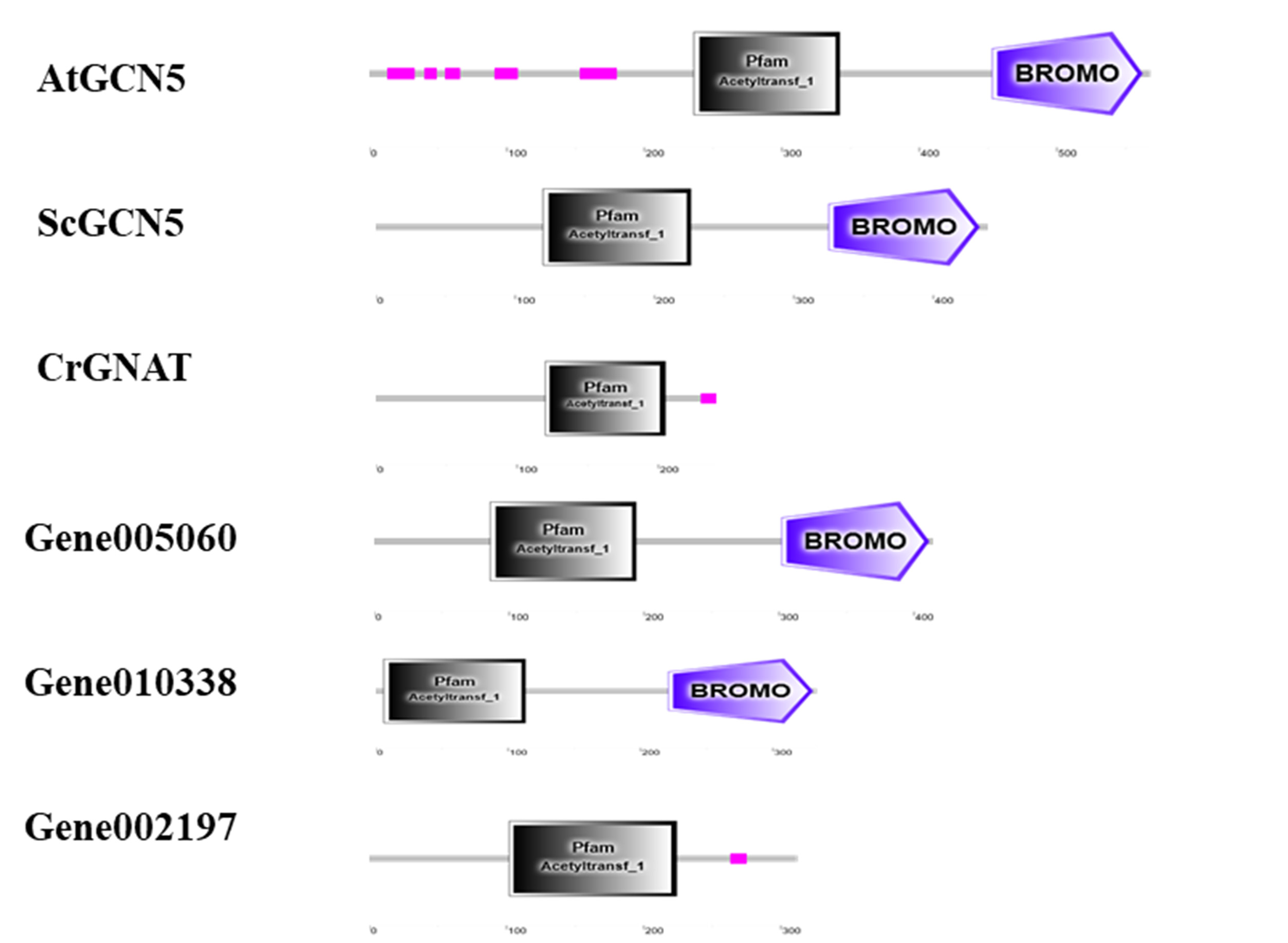

Supplement: Supplementary Figure S2 — The domain of GCN5 candidates from Haematococcus pluvialis and reported genes from Arabidopsis (AAK31318.1), Saccharomyces cerevisiae (NP 011768.1), and Chlamydomonas reinhardtii (XP_001693868.1) according to SMART analysis. [file Image_2.JPEG]
