# Supplementary material for: The Histone Acetyltransferase HpGCN5 Involved in the Regulation of Abiotic Stress Responses and Astaxanthin Accumulation in Haematococcus pluvialis
Source: Front Plant Sci. 2022 May 20;13:903764. doi: 10.3389/fpls.2022.903764 (PMC9163953; doi:10.3389/fpls.2022.903764)
Supplement: Supplementary Data 1 — The corresponding genome sequence of HpGCN5 downloaded from the BIG Data Center GSA Database accession no. PRJCA000614. [file Data_Sheet_1.docx]

**Supplementary Data 1**. The corresponding genome sequence of *HpGCN5* downloaded from the BIG Data Center GSA Database accession No. PRJCA000614. The yellow shaded nucleotides refer to the corresponding exons which from isolated *HpGCN5* cDNA sequence. The red shaded nucleotides refer to start and end site of coding region.

>*HpGCN5* genome sequence

GACACTAGGAGGACATCAGGACAAATGTAGGCGGGACTGAGCAATGGCTTACGACACAGACGAACGGTCGTCGAAAAGAGTCAAAGTGGAGGAACACGACGCGGGTTCTGAAAGGAATTGTCAGCTAGGGGATGTGCAGCAAGTAGAGCAGCGTACCGATGGCACACGAGTGTACAAAAGCGAGAAGACGGGCGCGTACTCTCAGCGCGAGGAGTTTTTAATAAGGCATGAAGCAGACGGCGACATCCGCTTCACCTATGTGGAAAACAACGGCGACCCGCATAACATGATGCACCTCATTAGCCTGAAGAACATATACTCCAAACAGCTTCCAAACATGCCCAAGGAGTACATCGTGCGCTTGCTGCTGCACTACTCCCACAAGTGAGTCCTACGGAGCACAACCCAGTGCATCTTAGTTCCCATGCCAGACAGGACTTCTTGATCCACCCTGCCTGGCCGCGCACCAGCTACACAGCCACACAAAATTGCAATCGCCCAACAACTACACCAACAACAACAATCCCAGCTGCAGACATGGCCATGGCCGCAGCCAAGGAGGGCAGGGAGCCATGTACTTATATAAATCGATGCGTTACCATGACGGGCTAAAGGGAACACAGAGCTGACGTGATACCAGACCAGCAGGCTTCCCCTCACCCCTCATGGGCCCCTCACAGTGGGATGCTTGCCGCTCCCTGACACTTTCCGGACAGCTTCCGAACAGCTTCCAGACAGCAAACCCCACGCAGTGGATGTGTCGCCATGCCTCCCGCCCCCCTGTACACAGTACCCCTGACAGTGGCCTCCTGGCAGCTTCCCGGCAGCTCCCTGCTCCCCCTGTGTTGGCCTGCAGGTCAGTGGTGCTGCTCAAGCGCAACGGGACTGTGATGGGGGGCATCACCTATCGTGCCTTCACGCCTCAGGTGCCTCATGCTGCCGCTGCCACTACTACCGCCGCTGCCACTGCTGCTGCTGCTGCTGCCACTGCTGCTGCTGCTGCCACTACTACCGCCGCTGCCTTTGCTGCTGCCTTTGCTGCCTTTGCTGCTGCTGCTGCTGCCGCTGCTCCCTCCTCTCACTGCTGCTGCGTAATGCTGCTTGACTTGTTGTTACCTCCTCCCTCCTCCTCCTCCCTGAAGCTGTGGCTGCTGCTGCAGCAGTCTGTGTTGGCGACGCCTGGTGTGTGTGTGTGCACAGGGGTTTGGTGAGATTGCGTTCTGCGCCATAGCTTCCAGTGAGCAGGTCAAGGGGTACGGCACGCGCCTCATGAACTACACCAAGGTGCGGGGAAAGGGAGGAGGCGAAGGAGCAGGAGGAAGAAGGAGGAGGAGGAGGAGGAGGAGAATCAGGGGGCGAGGGGGAGAGGAAGGGGACGCAGCAAGGGAGGAGGGTACGAGATGGGCGTGTGGGGAAGAGCATGCCAGCACGGGGGTAGATCATGGTCTCATCAAGGATTACAGTGGCTCTGATTGGCAGGCAGGCTGGCTGGCTGGCTGGCAGGCAGGCCAGTGGGTGGGTGGGTGGGCAAGTGGGTGGGTGTCTAGCACACCCTTGCATGGTCTGTGTGACCTGCCACAGGAGTACGCCGTGACCATGGACCGCCTGTCACACTTCCTCACCTATGCAGACAACAATGCGGTGGGCTACTTCACGAAGCAAGGCTTCACTAAGGAGATTACACTGGACAAGGCCAGGTGGGCTGGAGCAGGCCGGACCGGGTCTGTTGGGCTGGGCCGGGCATGTATAGCAGGGTGTTCTTTCCACAACACCATTCCTAGTCTTTACATATCTGGTGTGAATATATATATATATATATATATATATATATATATATATATATATATGGGTGTGGGATGACCTGGTCAAGCATAGCTGTGTCTCTCTCGGAAAAGTATAAAAGGTTTTCATTACCCAGCGTCCTTGCTGATCAGCTTCAGCCATGGATGCATGGCCTGTTGCCCTGCCACTACCTGAACCTACTGCAGAAGCCACCAATGCTGTCTACTGTGGCCACCTGCTCAGGTCCCAGACCTCTCCCCCTTCCTCTGCAGCCATCACTACAGCTGCCCACCAGGCACAGCGCCACCAGGCTCAGCGCCACCATGACACTGAGGCTGTGGCTGAGGCTGATGCTTAGGGCACCCTAGGCTGCTGCTGTTCATGAGAGACCTTGCACACCTTCTGCCCTGGACCCCCCTTCCTCCACACCCTTCTACCCTATCCCTGTACTGCCCCTAACCCCTCAGCCCCCCTCCCCTTCAAAACCTCCCACACTTTCCCCCCACTCGCTCGCCCACACACCCCGACCCTTCCCCCGCCCCCCCGACCCTCTAGCCCGTGTCCCCCCAGCCCCCTGGCCCTCTTGGCCCCACCCCCTGCCCCCTGCCCTCCGCCTGGCAGGTGGCAAGGCTACATCAAGGACTATGACGGCGGCACCCTGATGGAGTGTGTGATGCACCCTAAGCTGCGCCCCGCCCAGTTCCCCGCCCTGATCAGGGCGCAGAAGGCAGAGCTGGAGCGAATCATGCACGGCCTCTCCCACAGCCACATCATACACCCTGTAAGGGCAACACCAGCTGCTGGGGCGGGTCAGGTAGCCGGCAGGGTCTCTGCTGCACCAACCCTCTGTCGCCCCTGATCAAGACACACCACCTGATGGGCGGGTCAAGGCAGCTGGAGATCACGCACCAGCCCTCTGCTACCCCTGATAAAGTTGCTCCCCTCACCTGCCCTCCCCTGCTGTCCCATCCGCTGCCCTCCCCTGCCCTCCCCTCCCGTTGCACCTCCCCTGCCCGCCCCACCCCTGCAACACCCGTGTTGATGCTTTGCAGGGCCTGGCGCAGTTCAGGTCTGGGCAGCAGGCCTCAGTGCCAGTGGCCAGCATCCCCGGGGTGAAGGAGGCGGGCTGGTCAGAGGAGGCCGGACCCCCTCCCAGGTAAGGCCAGGCAGGCTGAGGCTGACAGCCACAACTGCAGCAGAGGGGATGAACTGGCAGCAACAGAGGGGCTGAGGGCGCTGTGTGGGTGACCACAGGCAGCAAGTTAGGAGGAAGGTTTGGAGGAAGGTTAGGAGGCAGGTGAGGAGGCAGGTGAGGAGGCAGGTGAGGAGGCAGGTGAGGAGGAAGGTTAGGGTGGGGCCAGGTGAGGAGGAAGGTTAGGGTGGGGCCAGGTGAGGCCTGACGAGGCAGGGAGCTCAGGTCCAGGTCCTGGGGGTAGGCATGAGCCTGCTGGCCCCTCCTACCCTCTAGCTGCCCTCTAGCTGCCCCTCTAGCTGCCCCTAACCTCTAGCCAGAATCGACTAGGAACGAGGGCAGGCTGTCTTGCTCCATGTCCAGGCACCCCTACTGCTTGCATGCTGCCTGCATGGTGGCCAGGTACCGGCTGCTGCTGAAGGAGGGGTCGGCTGAAGTCAACCCTGCAAGCCTCCACCGCTGGATGACCCTGGCCTTGCAGGAGGTGGGCTTGGGCTGCCTGCCGTCAAGCAGGAGCAGCAACAAAGGAAGCAGCACAGCATGGTGCCAGTATAGGCACAGGAACAGGCATGAGAAGGAGCAAAGGAAGACAGGGTGGCAGGCTCACCAGTGGAGGTGTCGAGGGTAGCCAGCATGGTGATGGTGGGTTTACACCGTAACAGCATCAGCCTGGTATGCTTGCTTGGCCAGCTTAGCCAGCATGGAAGGCACAGGGATTAGGGGAGGGACCTACAAAAGGGTATGTACCCTTGGGCCAACGCTGCCAGCAGTTGGCGTGCTCCTGCCCCGCCCTGCCCCCACACTGCCCTGCCCTGCCCCCTGGTCTCAACCCTGCCCCTGCCCCTGATGCCAACGCTTGCAGCACTCAACATGCCCCTGCCTCGCCCTTCCCCTGCCCCGCCCTGTCCCCACACTGCCCTGCCCCTGCTCCCTCCCCTGCCCCTCCCAGGTCCTACCCTGGCCCCTTGACTCTTCCCTGCCCTCCCCTTCCCCCCGCCCCTGCCACACCTCGACTCCAGCTACATAGCCCGTGCCCAGCGCCTGACCCCAACCCCTGACCGTGCCCCGCCGTGCCCCAGACCTGCCCAACCCCTGCCCCTGCCCTGCCCAACTCCACCGACCTCAGCTACAGGCCAGACAGGCTGTGTGCAGCGACCCTGCAGGTGCAGGGCCATGGTGACGCCTGGCCCTTCCTGCAGCCGGTCACCCGAGAAGAGGTGCCTGACTACCATGACATCATCAAGGTGTCTGCCTCACACCCTGACCTCACACCCTGACCTCACACCCTGACCTCACACCCTGACCTCACACCCTGACCTCACACCCTGACCTCACACCATGACCTCACACCATGACCTCACACCCTGACCTCACACCATGCTTCCTGATGTCAACTCAATCTGCAAGGTGTGTGATGTGTGGTTGGTGGGAGTTTGAGGCATACAGCGTCTACTCCTTGAGACGGCTACCCTCCTAATGCTGCACGCTGTGCTGTGCACCGGGTGCAGGACCCAGTGGACTTGTCCCTCATAGCCCGGCGCCTGGCAGGTCGCGTGTTCTACTCCCACCTAGATATCTTTCTGGCAGACTTCCGCCGCATGTTCAACAACTGCAGGTGCGTGAGGCATGGACCCAGCTGCACACCCGGCTCCCCAAACATATACACACATTGACACCCTGCCTGCCTGCCTGCCTGCCTGTGCTCTTGGCCCCCAGAACGTACAATTCCCAAGACACCATATACTACAAGCTCGCCAACAAGCTGGAGGCGCACCTGGATCAATGGGTGGCTGGGCACCTTGTCCACATGGGGTAGGCCATCAACCCGCTGAACCGTTGCCTCGCCCGACTGCGCAATACGCCCAACCCATCAACCCGCCCCGCCGCTACACCTGCCCGGACCGTATCCCGCCCGGCCGCCAACCCACCGCCCGGACCCACAGACCATGGCCCGTACCCTGCCCGGCCGTACCCGACGTTGCCTTTCCCCGCTCCGAGGGACGTTGGCGACGCTGCGGCCGTTGATCGGACGGTGCCATCGCTCGCTTGGTCCTTTACTATGCGCCCACCTCGGCCATACCCCGACATGCCCTGTACTCCCCACACCGCCCGCTATGAGGGAGGCTGGTGACATCGCGGTTGATCTCGCATCGTGCGCGACACCCCGGCCTTGGAGCGTACCAAGGGCGGGGCCTGCCCGGCCGTGGCTGTCGCGTAGTCGCAGTCGCTTGTTGGTACAC
